# Monitoring Biochemical and Structural Changes in Human Periodontal Ligaments during Orthodontic Treatment by Means of Micro-Raman Spectroscopy

**DOI:** 10.3390/s20020497

**Published:** 2020-01-15

**Authors:** Letizia Perillo, Fabrizia d’Apuzzo, Maddalena Illario, Luigi Laino, Gaetano Di Spigna, Maria Lepore, Carlo Camerlingo

**Affiliations:** 1 Dipartimento Multidisciplinare di Specialità Medico-Chirurgiche e Odontoiatriche, Università degli Studi della Campania Luigi Vanvitelli, 80138 Napoli, Italyfabrizia.dapuzzo@gmail.com (F.d.);; 2 Dipartimento di Scienze Mediche Traslazionali, Università degli Studi di Napoli Federico II, 80131 Napoli, Italy; 3 Dipartimento di Medicina Sperimentale, Università degli Studi della Campania Luigi Vanvitelli, 80138 Napoli, Italy; 4 CNR-SPIN, Istituto Superconduttori, Materiali Innovativi e Dispositivi, 80078 Pozzuoli, Italy; carlo.camerlingo@spin.cnr.it

**Keywords:** periodontal ligament, orthodontic tooth movement, Raman spectroscopy, proteins, CH_2_ and CH_3_ modes

## Abstract

The aim of the study was to examine the biochemical and structural changes occurring in the periodontal ligament (PDL) during orthodontic-force application using micro-Raman spectroscopy (μ-RS). Adolescent and young patients who needed orthodontic treatment with first premolar extractions were recruited. Before extractions, orthodontic forces were applied using a closed-coil spring that was positioned between the molar and premolar. Patients were randomly divided into three groups, whose extractions were performed after 2, 7, and 14 days of force application. From the extracted premolars, PDL samples were obtained, and a fixation procedure with paraformaldehyde was adopted. Raman spectra were acquired for each PDL sample in the range of 1000–3200 cm${}^{-1}$ and the more relevant vibrational modes of proteins (Amide I and Amide III bands) and CH${}_{2}$ and CH${}_{3}$ modes were shown. Analysis indicated that the protein structure in the PDL samples after different time points of orthodontic-force application was modified. In addition, changes were observed in the CH${}_{2}$ and CH${}_{3}$ high wavenumber region due to local hypoxia and mechanical force transduction. The reported results indicated that μ-RS provides a valuable tool for investigating molecular interchain interactions and conformational modifications in periodontal fibers after orthodontic tooth movement, providing quantitative insight of time occurring for PDL molecular readjustment.

## 1. Introduction

**Figure 1:**
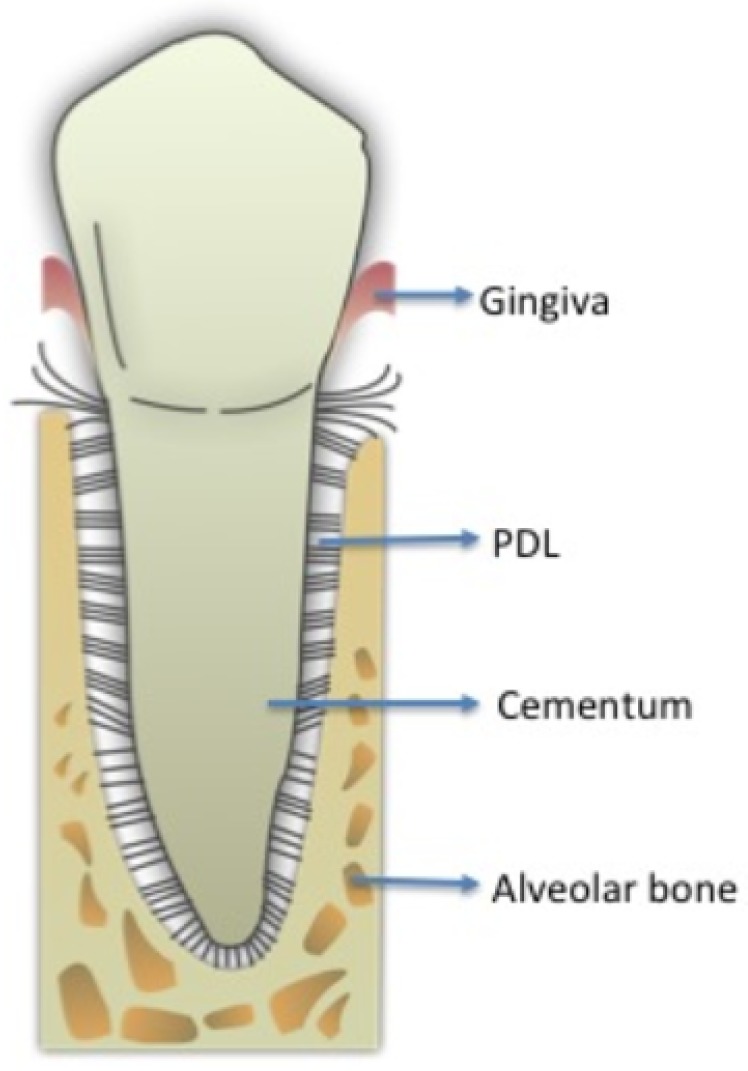
Periodontium with its components: gingiva, periodontal fibers (PDL) and cementum around the tooth root, alveolar bone supporting the tooth.

The periodontal ligament (PDL) is a membrane-like connective tissue interposed between the tooth root and alveolar bone (Figure 1). The medium thickness of the PDL is between 0.2 and 0.4 mm with its thinnest part located in the middle third of the root [1]. The most relevant components are collagen and elastic fibers associated with blood vessels and oxytalan tissues. PDL collagen is type I, with a percentage higher than 70%, type III at about 20% and a small number of others types of collagen fibers, i.e. type V that typically increases in the case of periodontal inflammation, and types VI and VII associated with blood vessels and epithelial-cell rests. The PDL also contains several cells such as fibroblasts, osteoblasts, osteoclasts, cementoblasts, mesenchymal cells, and nerve cell endings for proprioception, as well as fluids deriving from the vascular system [2,3]. Orthodontic tooth movement (OTM) alters the tissue structural properties at a cellular and molecular level. Mechanical stress from the application of orthodontic forces induces the activation of many mechanisms mediated by the release of several chemical substance cascades, allowing the transmission of signals from the extracellular matrix. These events lead to a gradual remodeling of tooth-supporting tissues during early OTM phases. Orthodontic force is optimal when characterized by maximal cellular response from the alveolar bone and periodontal ligament while maintaining the vitality of these tissue types [4,5,6]. The biological effects of an orthodontic force mainly depend on its intensity and area of PDL on which it is exerted. In fact, the compression of the periodontal fibers causes a phenomenon of “hyalinization” characterized by the disappearance and/or pyknosis of cell nuclei, and the disappearance of the collagen fibers tending to converge in a gelatinous substance. If the pressure applied on dental structures is too high there is an interruption of blood circulation in PDL followed by sterile necrosis and disappearance of the cellular component, thus the tissue remodeling occurs thanks to cells from contiguous areas, which invade the necrosis lacunae causing an indirect resorption. These mechanisms of hyalinization and resorption provoke an enforced delay in tooth displacement in the alveolar bone. Moreover, the presence of ischemic and inflamed areas in the PDL is the main cause of pain perceived by the patient during the orthodontic treatment [4,6]. Since 1962, Burstone considered OTM as consisting of three main phases: the initial phase, the lag phase, and the postlag phase. The initial phase, 24 to 48 h after the orthodontic force application, is characterized by evident immediate tooth movement due to its displacement in the PDL space, and after 48 h the real OTM starts after the alveolar bone remodeling occurring through the combined activity of osteoclasts and osteoblasts. After 20 days of force application (lag phase) there is little to no tooth displacement due to PDL hyalinization in the compression areas with no subsequent tooth movement until the complete removal of the necrotic tissues [4,5,6]. Detailed studies were performed on immunohistochemical, histological, and electron-microscopic of the human PDL during orthodontic treatment [6,7]. In particular, the outcomes revealed that the useful time points for orthodontic tooth movement monitoring should be set, at 2, 7 and 14 days after force application on teeth. The above-mentioned conventional methodologies are very time-consuming and labor-intensive, whereas optical diagnostic techniques showed some advantages [8]. Vibrational spectroscopies, such as Raman and infrared spectroscopies, can be effectively useful to analyze biological samples [9]. In particular, Raman spectroscopy is not affected by water presence and this is highly desirable in biological tissue analysis [10,11,12]. This technique was largely adopted for investigations on the conformational changes of the peptide backbone of protein fibers [13,14]. Stress application on chemical bonds determines some modifications in interatomic distances and consequently in the position of Raman bands due to the anharmonicity of the vibrational energy [10]. In recent years, micro-Raman spectroscopy (μ-RS) emerged as a non-destructive, non-invasive, very sensitive, and less time-consuming methodology for analyzing biological specimens, usually requiring simple or no special sample preparation when compared to traditional assays [12,15,16,17,18]. In our previous studies, a preliminary in vitro investigation on bovine PDL was performed to identify the most suitable sample-analysis experiment conditions [19]. Then, μ-RS efficacy in evaluating changes occurring in periodontal fibers after orthodontic-force application in human PDL samples was revealed [8]. Particular attention was focused on the analysis of the spectral regions related to the Amide I, Amide III and CH${}_{2}$/CH${}_{3}$ modes since the most important changes were foreseen in these spectral regions [8,19].

These results were preliminary and did not allow us to fully exploit the potential of μ-RS. Thus, the main purpose of this investigation was to extend the use of μ-RS by completely examining the biochemical and structural variations in obtained PDL samples from extracted premolars in orthodontic patients after a well-defined number of days of force application. Particular attention was paid to analysis of the spectral regions related to Amide I and III, and CH${}_{2}$/CH${}_{3}$ modes. Results of this investigation offer a particularly significant contribution in interpreting complex processes occurring after different time points of orthodontic tooth movement in human PDL and could be helpful in planning more accurate orthodontic treatments.

## 2. Subjects and Methods

### 2.1. Subjects

The age of the subjects ranged between 11 and 24 years and they needed orthodontic treatment with upper and/or lower first premolar extractions. They were consecutively recruited in the Orthodontic Program of the Multidisciplinary Department of Medical-Surgical and Dental Specialties at the University of Campania Luigi Vanvitelli, Naples, Italy. The research protocol complied with the tenets of the Declaration of Helsinki for the use of human tissues and approval for this study was granted by the Ethics Committee of the University of Campania Luigi Vanvitelli (N. Prot. 207). Informed consent was signed by each adult patient or minor patient’s parents. Inclusion criteria were full permanent dentition, good healthy periodontium with no evidence of bone loss on panoramic radiograph, no gingival inflammation and probing depth ≤3 mm in the whole dentition, full-mouth plaque score (FMPS) and full-mouth bleeding score (FMBS) were ≤20%. These indices were calculated as the percentage of tooth surface with supragingival plaque or bleeding, respectively, within 15 s of probing with a controlled ∼0.2 N force probe (Vivacare TPS Probe, Vivadent, Schaan, Lichtenstein). Exclusion criteria were previous fixed orthodontic treatment, systemic diseases, congenital deformities, as well as smoking and drug use in the month preceding the start of the protocol [20]. A total of 11 patients (7 females and 4 males, mean age 19.9±4.7 years) were selected (see Table 1). Complete pre-treatment records including panoramic and lateral skull radiographs, digital extra- and intra-oral photographs and dental casts from alginate impressions were collected. Since the periodontal ligament is not frequently collected during the current daily clinical practice due to low percentage of fixed orthodontic treatment involving extractions of healthy premolars the number of subjects is relatively small.

### 2.2. PDL Collection

**Figure 2:**
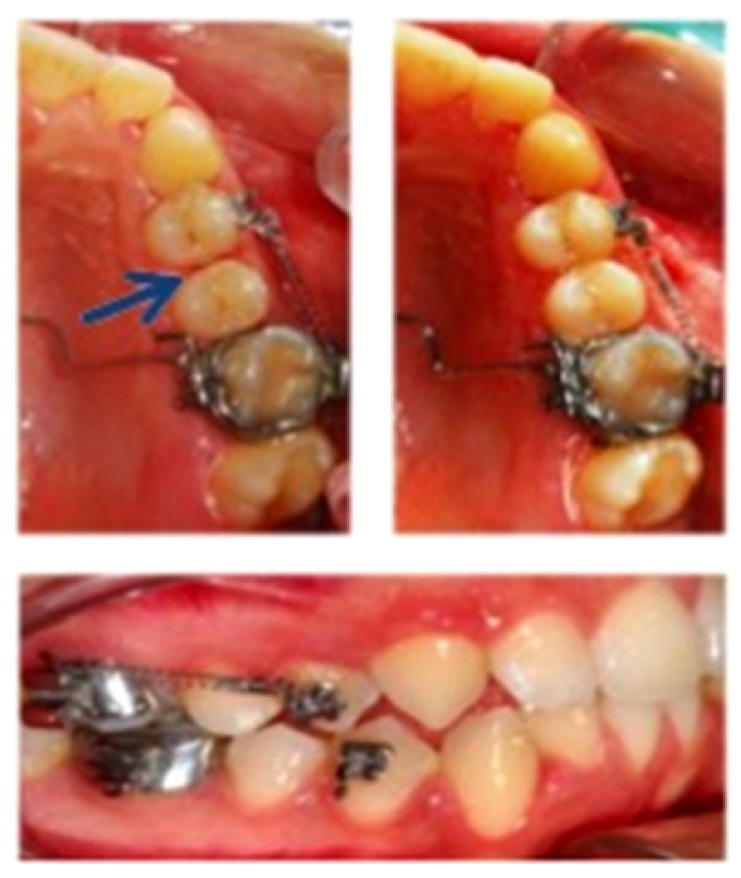
Performed interproximal reduction of upper first premolar to be extracted on the test side (blue arrow). closed-coil spring exerting ∼0.5 N force was attached between the premolar and molar and evaluated after 7 days of force application in these intra-oral photographs.

Orthodontic brackets with power pins (Victory Series Appliance System, 3M Unitek, California, USA) were used on the buccal surface of first premolars, and bands were applied on first molars. Before extractions, a closed-coil spring (Sentalloy 10-000-25, GAC International, Bohemia, NY, USA) exerting ∼0.5 N force was attached between first molars and premolars of the right upper and/or lower mid-arch considered test side, after 1-mm unilateral distal interproximal reduction of the first premolar with a diamond bur (859 L/010F type) (Figure 2). The left mid-arch was used as the control side.

Extractions of upper and/or lower first premolars were performed during the same dental visit on both sides with a clamp, to minimize damages to the periodontal fibers, as much as clinically possible. Patients were randomly assigned to three groups named A, B and C in which the upper and/or lower premolar extractions were scheduled after 2, 7, and 14 days, respectively, between force application and tooth extractions in order to take the three main phases of OTM into account. A PDL sample with dimensions of a few mm${}^{3}$ was scarified from the same side of the radicular tooth surface of the extracted premolars (mesial, or pressure side) using a 1-way lancet just after surgery. The same oral surgeon and orthodontist performed the previous steps to avoid any performance bias. For every patients and for different times of orthodontic treatments, 3 specimens were obtained from each PDL. Tissue samples were fixed in 4% paraformaldehyde (PFH) within three hours after the clinical scarification. PFH was removed by centrifugation (2000 rpm for 2 min). Samples were stored in ethanol until analysis [10,21,22].

### 2.3. Micro-Raman Spectroscopy

#### 2.3.1. Spectra Acquisition

Measurements with μ-RS were directly performed on the extracted PDL sample without any further manipulation. A Jobin–Yvon TriAx 180 Raman system (Horiba Scientific, Osaka, Japan) with a λ=633 nm excitation light wavelength (He-Ne laser), was used for PDL sample characterization. The Raman signal was dispersed by a grating of 1800 grooves/mm and collected by a liquid N${}_{2}$ cooled Couple Charge Detector (CCD). The spectral resolution was 4 cm${}^{-1}$. The excitation light of the 17 mW He-Ne laser was directed on the sample using a 100× optical objective on a spot area of about 2 μm${}^{2}$. Three different spectra were obtained from each portion of PDL tissues at different locations. Acquisition time was typically to the order of a few minutes, ranging between 60 and 300 s and was properly chosen for each spectrum in order to minimize fluorescence background [23].

#### 2.3.2. Data Treatment

Background and noise components were numerically reduced by using a wavelet-based algorithm on the whole experiment dataset. This numerical-data treatment provided an efficient automatic method for weak and noised Raman signal refinement [16]. Similar to more conventional numerical filter methods based on Fourier transform analysis, the algorithm based on wavelets uses specific mathematical functions instead of the sinusoidal ones, allowing multi-resolution local analysis of the spectrum, at different signal-variation scales. After signal decomposition, the spectrum could be reconstructed by an inverse process, removing low- and high-scale components correlated with background and random-noise contributions, respectively. The above-described data analysis was implemented by using MATLAB 6.5 program (MathWorks Inc., Natick, MA, USA). Spectral signals were decomposed by using the “bior 6.8” wavelet family of biorthogonal functions up to the n = 8 level of scaling and reconstructed with 5th and 6th levels of detail components [16]. After wavelet-data treatment, the integral of the signal was calculated over the spectral range of interest and the signal was normalized to 1 to allow direct comparison of data or the average of spectra collected under the same experiment conditions.

#### 2.3.3. Spectral Deconvolution Procedure

An evaluation of the main Raman modes in the measured signal could be achieved by performing a deconvolution of the spectrum in elemental components. In this study, a best fit of the data was performed by comparing the spectra with a model based on the overlap of a set of mixed Lorentzian/Gaussian peaks. We assumed as fitting parameters the function kind (Lorentzian or Gaussian) and its spectral positions, intensities and widths after minima localization of the second derivative spectrum, which corresponded to the positions of peaks within the band. In particular, second-derivative spectra were obtained with Savitsky–Golay derivative function algorithm for a seven data point window [24]. The best fit was done using a library routine of GRAMS/AI (2001, Thermo Scientific TM, Waltham, MA, USA). Fit convergence was evaluated by the $\chi^{2}$ parameter with the Levenberg–Marquardt nonlinear least-square method. Particular attention was devoted to the Amide I (1550–1750 cm${}^{-1}$), Amide III (1200–1350 cm${}^{-1}$) bands and the CH${}_{2}$/CH${}_{3}$ modes (2800–3000 cm${}^{-1}$), where significant variations were expected.

#### 2.3.4. Statistical Analysis

Numerical data were expressed as mean ± Standard Deviation (SD). Data were statistically analyzed by using one-way ANOVA test and *p* values less than or equal to 0.05 were considered to be statistically significant (* *p* ≤0.05; ** *p* ≤0.01; *** *p* ≤0.001). The statistical analysis was performed by using Origin software (Version 9.0, OriginLab Corporation, Northampton, MA, USA).

## 3. Results

**Figure 3:**
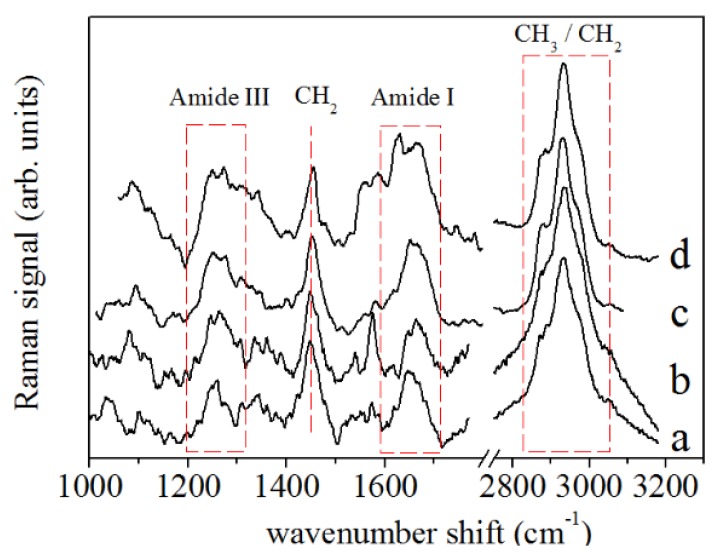
Raman spectra of PDL samples for (**a**) untreated case (control), (**b**) after 2 days, (**c**) 7, and (**d**) 14 days of OTM. Main Raman bands due to protein and CH${}_{2}$/CH${}_{3}$ contributions are highlighted.

**Figure 4:**
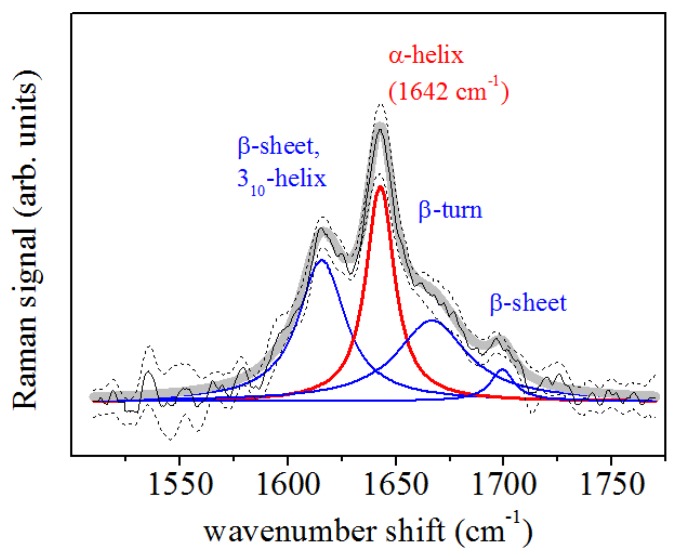
Deconvolution of PDL Raman spectrum in the Amide I region. Data were obtained by averaging the Raman response of control samples. The α-helix component (red line) and other main components (blue lines) of the Raman band are indicated.

A representative Raman spectrum obtained by averaging all spectra from the control PDL samples is shown in Figure 3 (spectrum a) and it is compared with the average spectrum of the PDL samples after 2 (Figure 3, spectrum b), 7 (Figure 3, spectrum c) and 14 days (Figure 3, spectrum d) of in vivo application of ∼0.5 N force by an orthodontic closed-coil spring. In particular, bands at 1600–1700 cm${}^{-1}$ (Amide I mode) and 1200–1350 cm${}^{-1}$ (Amide III mode) were assigned to protein vibrations, while the broad band centered at 2930 cm${}^{-1}$ was assigned to the CH${}_{2}$ and CH${}_{3}$ bond vibrations [25]. Another Raman mode, at about 1450 cm${}^{-1}$ was assigned to CH${}_{2}$ scissoring Raman mode and protein components. In Figure 4, the deconvolution of Raman signals concerning data collected from a PDL control sample for the spectral region of Amide I is reported. This band consisted of the major components that are generally related to the secondary structure of the protein. This band allowed us to obtain information on collagen which is the main PDL component. The mode assigned to the α-helix mode, at about 1640–1645 cm${}^{-1}$, dominates the band.

**Figure 5:**
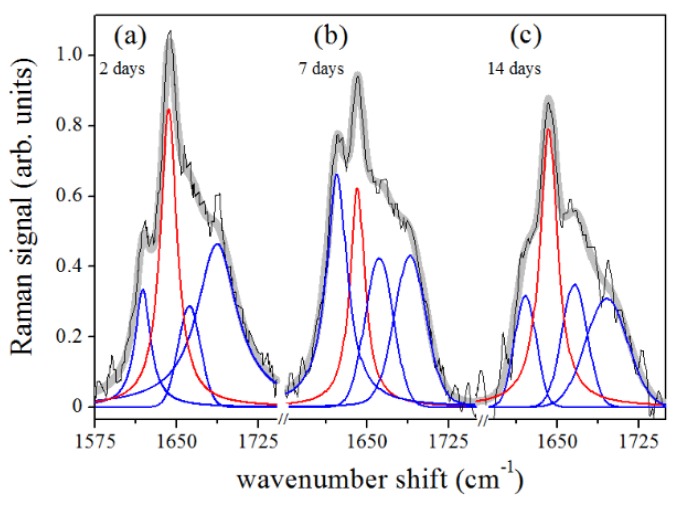
Deconvolution of PDL Raman spectra in Amide I region. The data were obtained by averaging the Raman response of samples extracted after (**a**) 2 days, (**b**) 7 days, and (**c**) 14 days of OTM. α-helix component, red line; other main components, blue lines.

Further peaks occurring in the band were centered at about 1620 cm${}^{-1}$, 1668 cm${}^{-1}$, and 1680 cm${}^{-1}$ and were assigned to β-sheet or collagen $3_{10}$-helix, β-turn and β-sheet secondary structure conformations, respectively [25,26,27,28,29]. The results of the peak centers (in cm${}^{-1}$) of the main Raman modes occurring in the Amide I region at different treatment times of sample collection are reported in Table 2. Figure 5a–c shows the spectral deconvolutions of the Amide I regions of the PDL Raman signals after 2, 7, and 14 days of force application. Each spectrum was fitted with a mixed set of Gaussian and Lorentzian (for the α-helix mode)-peak functions, as previously described.

**Figure 6:**
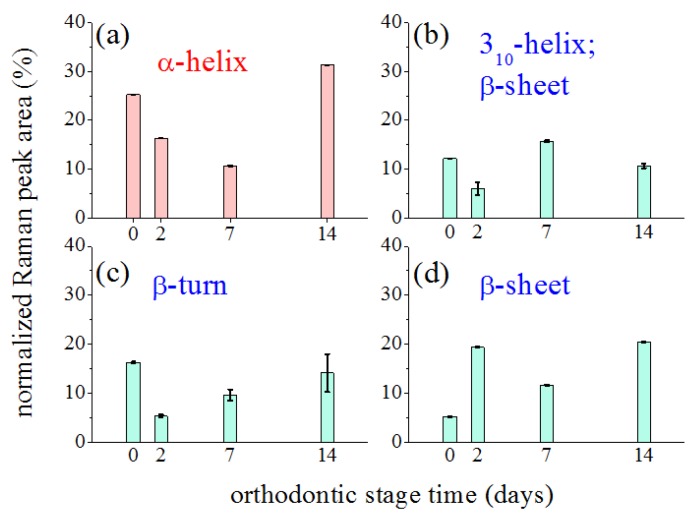
Changes induced by orthodontic force application on the Raman signal intensity of the main components of the Amide I band. Reported data refers to percentage of normalized areas of considered components in whole Amide I band area, assumed equal to 1. Black bars indicated error of area value determination.

Figure 6 outlines the influence of the orthodontic-process length on the characteristics and the relevance of the main modes contributing to the Amide I band by plotting their relative spectral area as a function of the treatment time. Modes were considered relative to the α-helix in the 1641–1643 cm${}^{-1}$ (Figure 6a), $3_{10}$-helix in the 1617–1621 cm${}^{-1}$ (Figure 6b), β-turn in 1661–1668 cm${}^{-1}$ (Figure 6c), and β-sheet in 1687–1695 cm${}^{-1}$ range (Figure 6d) [27,29]. The reported peak areas were normalized to the total area of the Amide I region, to quantify their relative contribution to the Amide I band.

The Amide III region can also offer interesting information about secondary protein structures [30,31,32]. The spectral band in 1270–1310 cm${}^{-1}$ range is generally assigned to the α-helix secondary structure. Assignments for random coil (1240–1270 cm${}^{-1}$) and β-sheet (1230–1250 cm${}^{-1}$) were also determined [30,32]. Table 3 summarizes the obtained results from spectrum deconvolution in terms of Lorentzian curves of the Amide III region. The α-helix band was centered at about 1307 cm${}^{-1}$. This value did not change significantly in the different phases of orthodontic treatment, as in the case of Amide I (see Table 2). Slight hardening, dependent on the treatment time, was instead featured by the random coil and β-sheet modes. Differently from the case of Amide I, further analysis of the relative spectral area as a function of treatment time was not performed because the Amide III region has a relatively complex structure due to the presence of additional components that cannot be assigned to Amide [31].

**Figure 7:**
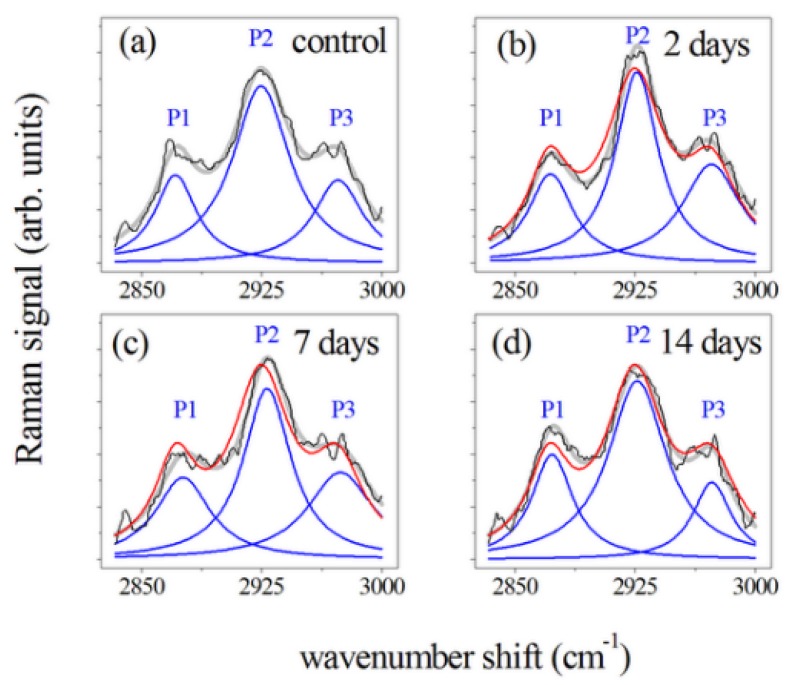
Deconvolution of the Raman spectra of PDL samples in the 2800–3000 cm${}^{-1}$ spectral region. Measured spectra, black lines; main P1, P2 and P3 Lorentzian components of the Raman band, blue lines. Gray lines refer to the fit of the experimental data resulting from the deconvolution process. Data were obtained by averaging the Raman response of PDL samples from (**a**) control, and samples after (**b**) 2, (**c**) 7, and (**d**) 14 days of orthodontic force application. All the band areas were normalized to 1 and spectra in (**b**–**d**) were compared with Raman response (red line) of controls (**a**).

**Figure 8:**
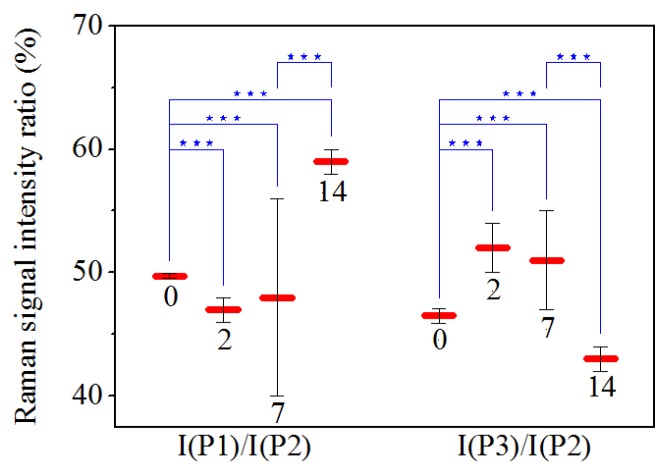
Comparison of mean values of Raman signal intensity ratios from control samples (0) and samples after 2, 7, and 14 days of OTM. The numerical values are expressed as the mean ± SD. The asterisks indicate that a significant difference between ratios occurred at * *p* ≤0.05; ** *p* ≤0.01; *** *p* ≤0.001.

Figure 7 shows the Raman spectra of the CH${}_{3}$ and CH${}_{2}$ modes [28,29,33] for PDL samples collected (Figure 7a) before, and after (Figure 7b) 2, (Figure 7c) 7, and (Figure 7d) 14 days of orthodontic force application. These spectra were normalized in comparison to the total area and obtained by averaging spectral features of samples from subjects with a similar clinical history as the control and PDL sample. A large peak at 2930 cm${}^{-1}$ (indicated by P2 in Figure 7) was predominant in this region of the spectrum. This mode is generally assigned to CH${}_{3}$ symmetric stretching vibration. The two peaks occurring in the band, at 2875 cm${}^{-1}$ (P1) and 2970 cm${}^{-1}$ (P3), were also related to C-H bond modes, and, more precisely, to CH${}_{2}$ asymmetric and CH${}_{3}$ asymmetric stretching modes, respectively [16,28,29,33,34]. Some changes occurred in this region of the spectra after orthodontic treatment. Intensities of the P1 and P2 peak were initially enhanced, after 2 days of force application, decreased after 7 days of treatment and then showed an enhancement in the last period of force application. Different behavior was observed for P3 peak after 14 days of OTM. In the spectra of PDL samples, the I(P1)/I(P2) and I(P3)/I(P2) intensity ratios also changed comparing to the control values (see Table 4). Statistical analysis indicated that ratios significantly change (*p* ≤0.001) in almost all cases as shown in Figure 8. For data referring to 2 and 7 days of treatment statistical analysis showed that they are not significantly different.

## 4. Discussion

The reported investigation aimed to assess changes in obtained PDL samples from extracted premolars in orthodontic patients using μ-RS. Several differences were recorded in periodontal tissue after different time points of orthodontic-force application. The accurate examination of vibrational modes showed in the PDL Raman spectra collected after 2, 7, and 14 days of OTM showed two principal regions: Amide I and CH${}_{3}$/CH${}_{2}$. They were both mainly correlated with the collagen structure while some contributions in the CH${}_{3}$/CH${}_{2}$ region also could be associated with tissue lipid components. Figure 5 underlines the large variation in the Amide I region of the spectra collected at different time points of OTM. In particular, the α-helix Raman mode and the whole Amide I band decreased in intensity when compared to the control PDL Raman spectra. The remaining component modes of Amide I became broader and stronger with respect to the α-helix mode, which is essentially composed of a hierarchical structure that could reach an assortment of parallel H-bonds at higher molecular level starting from individual hydrogen (H) bonds. The α-helix has a single-stranded conformation forming a spring-like protein structure with 3–4 H-bonds per turn. In the case of collagen, the α-helix assumes a peculiar triple helix configuration. This structural arrangement is considered the most effective bond arrangement for thermodynamical and mechanical stability providing high elasticity and large deformation capacity. The β-sheet structure could instead be considered a partially unfolded α-helix structure with a combination of broken and intact H-bonds along the filament axis [35]. In our previous study [8], the signal in the Amide I region decreased after two weeks of orthodontic-force application. We observed an intensity decrease of the mode assigned to the α-helix secondary structure, reaching a change of more than the 75% compared to the initial values after orthodontic treatment of 14 days. The initial stage of the process is the most critical and relevant change of Raman response intensity was observed during the first 48 h of tooth movement. This effect was partially compensated in the next days of the orthodontic treatment, during the first week, when a little recover seemed to occur. Explication can be found in the properties of the helical protein structure and its response to strain forces, widely considered in the literature both theoretically [36] and experimentally [37,38]. Due to the peculiar helical structure, enhanced resistance to mechanical strain is a protein feature that prevents the occurrence of breakages and large bonding deformations. A reversible breaking of H-bonds is the basic mechanism of protein unfolding of the α-helix. These processes allow the dissipation of mechanical energy through the formation of specific regions and avoid the breakage of stronger molecular bonds [37]. The application of a strain leads to the necessary tissue stress to begin the modification of the α-helix structure or the transformation from the α-helix to the β-sheet conformation. This is a result of the above-discussed unfolding-refolding processes, that promotes a hydrogen relocation, and an increase of disorder [8,38]. This effect was proven by Raman spectroscopy monitoring single keratin fibres from hair during a straining process [37]. In particular, the authors found a depression of the Raman signal of the α-helix component in the Amide I band together with a continuous increase of the β-sheet modes with ongoing strain. These outcomes were comparable with the obtained results in our PDL samples (Figure 5 and Figure 6). The OTM induced some changes in the secondary protein structure if a decrease of the α-helix modes occurred in the tissue. This modification mainly happened after 2 days of force application with a general decrease and broadening of the Amide I band. After 7 days, readjustment of the protein structure with bond relocation and formation of H atoms occurred. After 14 days, the Raman spectra in the Amide I band reported a larger β-sheet and/or random disorder components than ordered α-helix ones, probably due to the protein denaturation with additive mechanisms (Figure 6).

Concerning the 2800–3000 cm${}^{-1}$ region (mainly due to CH${}_{3}$ and CH${}_{2}$ modes) [28,33], an increased intensity of the peak at 2930 cm${}^{-1}$ was mainly observed after two days of treatment. To explain these results, two main events occurring at the biochemical level in the compression sites during orthodontic-force application have to be considered i.e., local hypoxia and mechanical transduction. Local hypoxia leads to the increased expression of some interleukins (IL), such as IL-1, IL-6, IL-8, tumor necrosis factor (TNF), and vascular endothelial growth factor (VEGF) in PDL fibroblasts, whereas mechanical transduction is due to the physical strain, stimulating PDL cells to generate growth factors, prostaglandin E2 (PgE2) and chemokines [39]. From these molecules, PGE2 is one of the most deeply inspected osteoclastogenic mediators being the earliest marker of bone modeling. Prostaglandins (PGs) are part of the eicosanoid family (oxygenated C20 fatty acids) produced by many cells in PDL tissues. They are the first lipid messengers and are synthesized from arachidonic acid (AA) by cyclooxygenase (COX) enzymes that are already expressed or produced in response to cell-specific stimuli, trauma, or signaling molecules [40]. Other principal products of arachidonic metabolism are leukotrienes that were also shown to increase their number around the teeth undergoing orthodontic movement. This may explain the observation that a non-steroidal anti-inflammatory drug in an animal model decreases the osteoclast numbers but not the tooth movement suggesting the occurrence of some overlap and redundancy between the different pathways. The inhibition of leukotriene production results in the reduction of tooth movement [41]. In the literature, the different roles of PGE2 were described. For example, Raisz et al. [42] reported that PGE2 plays an important role in the replication, differentiation, and fusion of osteoclasts. PGE2 has a double capacity: it stimulates the production of receptor activator nuclear factor-kB ligand (RANKL) and inhibits osteoprotegerin (OPG) production; moreover, when a compressive static force is applied, the RANKL is expressed in PDL cells [42,43,44,45,46]. These outcomes confirm that PDL cells might present a regulatory role in alveolar bone resorption in the compression area during OTM. Kang et al. [46] examined the focal adhesion kinase (FAK) activation as a mechanoreceptor in human PDL cells and the resulting production of PGE2 in vitro. In that study, PDL cells were collected from premolars extracted for orthodontic treatment. Then, compressive static force was applied to the PDL cells in-vitro for different times (0.5, 2, 6, 24, and 48 h). Those results showed that compressive stimulation increased the level of phosphorylated FAK, PGE2 production, and up-regulated the cyclo-oxygenase-2 mRNA mainly after 48 h of the force application. Toledo et al. [47] stimulated parasite Trypanosoma cruzi with AA the, cultivated in peritoneal macrophages from uninfected C57BL/6 mice, to demonstrate that lipid body organelles are produced after both host interaction and exogenous AA stimulation. Raman spectroscopy and MALDI mass spectroscopy were used to demonstrate the increase of the Raman bands at 3015 and 2929 cm${}^{-1}$ representing AA lipid spectra. Biologically active eicosanoids (PGs) are derived from AA, so the authors investigated if they are produced by PGs from the AA. We suggest that the intense and broad peak around 2930 cm${}^{-1}$ observed in the PDL spectra of the sample after 48 h of treatment, correlated with the AA by Toledo et al. [47], could be connected to the increase of PGE2, seen by Kang et al. [46] after 48 h of a treatment with a compressive force. The observed properties are consistent with the previous considerations about the Raman signal intensity dependence on orthodontic process time. Thus, μ-RS revealed great potentiality in reporting the specific molecular fingerprinting of PDL samples, but further investigations are required to illustrate a direct correlation between secondary protein structure in the PDL samples showed by Raman spectra and the tertiary structure of the molecules involved in the tissue remodeling during OTM.

## 5. Conclusions

The present investigation of PDL samples using μ-RS after orthodontic-force application allowed a useful inspection of molecular arrangements and conformational changes in periodontal fibres after different time points of orthodontic-force applications. In particular, the Raman response in the Amide I spectral region showed the important role of α-helix conformation to limit damages due to external forces and to avoid collagen fibre breaks by configuration changes featured by the Raman signal from $3_{10}$-helix and β-sheet secondary structures. Clear restoring mechanisms in PDL samples were shown after more OTM days. μ-RS provided quantitative evaluation of time occurring for the readjustment of PDL tissues at the molecular level. μ-RS response analysis in the 2800–3000 cm${}^{-1}$ spectral region was also discussed and confirmed the tissue changes mainly after two days of orthodontic force application. Thus, μ-RS provided valuable insight into molecular interchain conformational changes in the PDL subjected to orthodontic forces and offer a particularly significant and unique contribution in interpreting the complex processes occurring after different time points of orthodontic force applications into the human periodontal fibers. The results also highlighted the challenges of Raman spectroscopy applications in monitoring the periodontal status at the biochemical level in subjects undergoing orthodontic treatment and consequently in providing a better customized treatment for each patient.

## Figures and Tables

**Table 1:** Demographics of subjects included for PDL collection.

GROUP	SUBJECT CODE	SEX	AGE (years)
A (2-day OTM)	a	M	20.9
	b	M	25.9
	c	F	18.0
	d	M	20.8
B (7-day OTM)	b	M	25.9
	e	F	24.1
	f	F	13.8
	f	F	13.8
	g	F	28.4
C (14-day OTM)	h	M	26.7
	c	M	18.0
	i	F	17.2
	i	F	17.2
	j	F	20.2
	k	F	3.9
		**Mean AGE ± SD**	**19.9 ± 4.7**

**Table 2:** Raman Amide I band deconvolution of PDL samples at different stages of orthodontic treatments. Bold fonts, Gaussian-shaped peaks; normal fonts, Lorentzian peaks.

Orthodontic Treatment (days)	α-Helix (cm${}^{-1}$)	$3_{10}$-Helix; (cm${}^{-1}$)	β-Turn (cm${}^{-1}$)	β-Sheet (cm${}^{-1}$)
0	1642	1617	1668	1695
2	1643	1619	1662	1687
7	1641	**1622**	**1661**	1690
14	1642	**1621**	**1666**	**1695**

**Table 3:** Amide III band deconvolution of PDL samples at different stages of orthodontic treatments. Lorentzian functions were used in the fitting procedure.

Orthodontic Treatment (days)	α-Helix (cm${}^{-1}$)	Random Coil (cm${}^{-1}$)	β-Sheet (cm${}^{-1}$)
0	1309	1258	1243
2	1307	1265	1245
7	1310	1266	1247
14	1307	1273	1252

**Table 4:** Deconvolution of Raman spectra of PDL samples in 2800-3000 cm${}^{-1}$ region. Raman signal intensities for main components (P1, P2, and P3) and ratios of P1 and P3 component intensities compared to P2 one (in percentage) reported at different stages of OTM. Raman band areas were normalized to 1.

Orthodontic Treatment (days)	I(P1) $\times 10^{-3}$ (a. u.)	I(P2) $\times 10^{-3}$; (a. u.)	I(P3) $\times 10^{-3}$ (a. u.)	I(P1) /I(P2) (%)	I(P3)/ I(P2) (%)
0	5.02 ± 0.03	10.10 ± 0.03	4.70 ± 0.05	49.7 ± 0.2	46.5 ± 0.6
2	5.07 ± 0.06	10.90 ± 0.06	5.6 ± 0.1	47 ± 1	52 ± 2
7	4.7 ± 0.8	9.7 ± 0.4	5.0 ± 0.2	48 ± 8	51 ± 4
14	6.00 ± 0.03	10.20 ± 0.03	4.40 ± 0.05	59 ± 1	43 ± 1

## Abbreviations

The following abbreviations are used in this manuscript:

AA	Arachidonic Acid
COX	Cyclooxygenase
FAK	Focal Adhesion Kinase
FMBS	Full-Mouth Bleeding Score
FMPS	Full-Mouth Plaque Score
IL	Interleukins
μ-RS	micro-Raman Spectroscopy
OTM	Orthodontic Tooth Movement
PDL	Periodontal Ligament
PFH	Paraformaldehyde
PG	Prostaglandin
PgE2	Prostaglandin E2
RANKL	Receptor Activator Nuclear Factor-kB Ligand
TNF	Tumor Necrosis Factor
VEGF	Vascular Endothelial Growth Factor
